# Editorial: Learning interventions and training: providing support during health emergencies

**DOI:** 10.3389/fpubh.2025.1597628

**Published:** 2025-04-25

**Authors:** Heini Utunen, Matthew Strehlow, Jamie Sewan Johnston, Qiang Zhang, Jane Noyes, Bruce Struminger

**Affiliations:** ^1^World Health Organization, Geneva, Switzerland; ^2^Center for Health Education, Stanford University, Stanford, CA, United States; ^3^Department of Emergency Medicine, School of Medicine, Stanford University, Palo Alto, CA, United States; ^4^School of Social Development and Public Policy, Beijing Normal University, Beijing, China; ^5^School of Medical and Health Sciences, Bangor University, Bangor, United Kingdom; ^6^ECHO Institute, University of New Mexico, Albuquerque, NM, United States

**Keywords:** health, emergency, response, learning, health worker, health worker capacity building, adult education

In an increasingly globalized and interconnected world, health emergencies have grown in scale and complexity. From humanitarian crises to natural disasters and emerging epidemics, our goal is simple: to advance the wellbeing of the world's people and keep them safe in emergencies.

**Figure 1:**
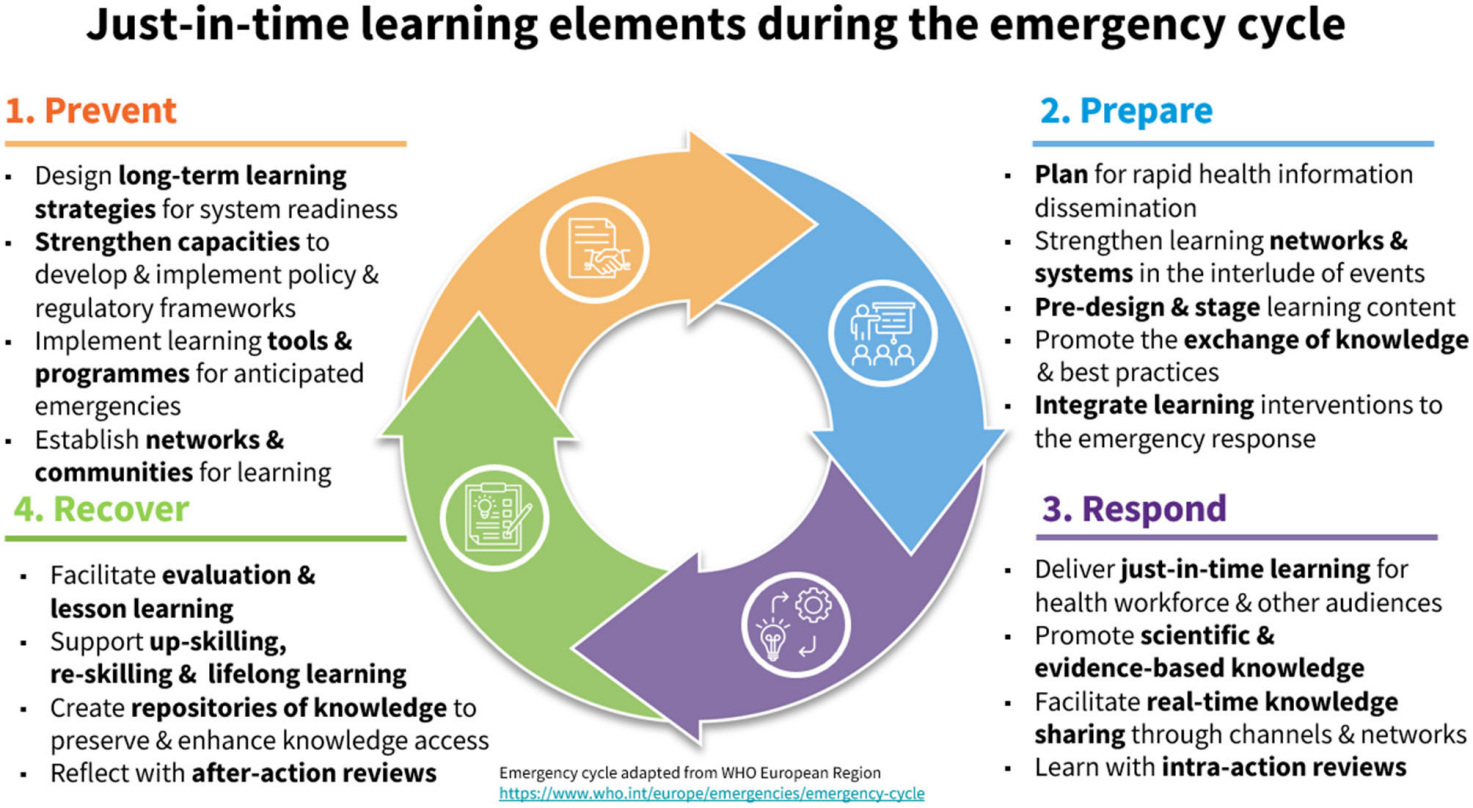
Just-in-time learning elements during the emergency cycle.

As part of this effort, a high priority has to be placed on providing timely and equitable access to knowledge, science and evidence. Just-in-time learning is about delivering specific training to support professionals with the information, knowledge and skills they need for emergency situations that impact public health (see Figure 1). Providing just-in-time learning can enable policymakers, health professionals and emergency workers to proactively mitigate the effects of health hazards.

The COVID-19 pandemic reaffirmed the importance of just-in-time learning on a massive scale. Since the beginning of the pandemic, people sought trusted information and knowledge to protect themselves, their families and their communities from this new and emerging health threat. The World Health Organization's OpenWHO.org learning platform saw a surge in demand, growing from thousands of enrolments to more than 9 million across free online courses on the pandemic and public health topics.

Affected communities must also be empowered to ensure information reaches the people who need it most, as evidenced in the recent World Health Organization's just-in-time guidance (1). Drawing on available evidence and operational insights, the guidance provides practical strategies to empower health professionals, policy-makers, emergency responders, volunteers and communities. Production of the guidance also identified large gaps in what we know and need to know to better respond to health emergencies where just-in-time learning is required. This Research Topic provides a mechanism for researchers and practitioners to publish additional and much needed evidence.

The articles in this Research Topic describe how public health and learning professionals have provided learning interventions to strengthen health emergency response by addressing and mitigating the impacts of infectious threats, natural hazards, humanitarian crises and armed conflicts. In all of these contexts, it has been vital to provide learning opportunities to support health professionals, emergency responders and the public with life-saving information, tools and skills to respond effectively.

More specifically, the 16 articles in the Research Topic “*Learning interventions and training: providing support during health emergencies*” focus on various strategies for enhancing healthcare workers' capacity to manage health emergencies. They cover themes such as continued education, just-in-time learning for healthcare workers, the evolving role of innovative, digital tools and mobile platforms and virtual simulations. The breadth of research across these areas provides diverse insights through different contexts, geographical focuses and explorations of specific health crises and interventions.

New frameworks and initiatives are identified that can support further interventions in future health emergencies (D'Andrea, Fadul, Struminger et al.; Mills et al.; Mayigane et al.). D'Andrea, Fadul, Struminger et al. and D'Andrea, Fadul, Dery et al. highlight the benefits of virtual learning in low-resource and conflict-affected settings and provide a framework for anticipatory digital learning. Strehlow et al. demonstrate the potential of massive open online courses (MOOCs) to rapidly provide access to emerging medical knowledge during public health emergencies particularly in high- and middle-income countries. Barnadas et al. explore the usefulness of knowledge sharing sessions specific for the laboratory workforce held between 2020 and 2023. Elhakim et al. highlight the existing frameworks provided by the WHO, including through voluntary tools provided under the International Health Regulations' Monitoring and Evaluation Framework, and their benefits to enhancing resilience and country readiness for health emergencies. Balde et al. explore the lessons learned from the emergency medical teams' initiative noting a need for enhanced training and capacity-building programs. Pandya et al. highlight the usefulness of simulation exercises to support low-cost and low-resource learning for disaster preparedness.

Common challenges to learning and knowledge transfer are cited across these articles including internet connectivity, different contexts, resource limitations, and additional training needs (Barnadas et al.; Bonkoungou et al.; Southworth et al.; Zhu et al.; Reynolds et al.; Balde et al.). Common benefits to the successful deployment of learning interventions and training to provide support during or for future health emergencies are similarly uncovered. These include cost-efficiency and adaptability of online learning, more timely responses, greater access and equity among learners, and positive responses of learners to the relevance and usefulness of the learning itself (Barnadas et al.; Bonkoungou et al.; Tian et al.; Walldorf et al.; Zhong et al.).

Sharing knowledge and enhancing just-in-time learning will make a difference in the health crises of the future. We hope to encourage more health emergency response institutions and professionals to invest in capabilities for just-in-time learning and continue producing evidence of this critical work, acknowledging it is a huge task amidst the hours of response.
